# Increasing safe clinical spaces and the efforts of clinical research for uninsured and underinsured LGBTQIA2+ patients: A case study of the Rainbow Clinic – a student-run free LGBTQIA2+ clinic

**DOI:** 10.1017/cts.2023.641

**Published:** 2023-10-09

**Authors:** Gabriel Alexander Lee, Jessica Fritter, Courtney DuBois Shihabuddin

**Affiliations:** 1 The Ohio State University College of Medicine, Columbus, OH, USA; 2 The Ohio State University College of Nursing, Columbus, OH, USA

**Keywords:** LGBT+ health, free clinic, underinsured, HIV/AIDs, trauma-informed care, gender affirming care, LGBT+ research

## Abstract

LGBTQIA2+ patients often experience discrimination and hostility in healthcare spaces. Negative perceptions of healthcare can contribute to poor health outcomes in LGBTQIA2+ patients. This population is rarely included in clinical trials through a lack of inclusion in study protocols, informed consent, and trials not addressing their needs and demographics. Many clinical institutions have created LGBTQIA2+-specific clinics; however, few have successfully developed a free clinic dedicated to this population. A Rainbow Clinic was formed at an established student-run free clinic, utilizing the existing infrastructure. Dissemination of this clinic’s creation can help others replicate similar initiatives.

## Introduction/Problem

LGBTQIA2+ (Lesbian, Gay, Bisexual, Transgender, Queer, Intersex, Asexual, Two-Spirit) patients often experience both overt and covert forms of discrimination and hostility in healthcare spaces. While overt aggression against LGBTQIA2+ patients has declined due to hospital-administered diversity and/or implicit bias training, many patients experience covert hostility known as microaggressions. Microaggressions taint LGBTQIA2+ patients’ perceptions of healthcare and their overall healthcare experience [1]. These microaggressions in healthcare spaces can take the form of innocent mistakes and include not using the pronouns a patient goes by and deadnaming [2], where an individual is called a name, they no longer use such as a birth name for some trans and non-binary patients. While these actions are often unintentional, they contribute to LGBTQIA2+ patients’ negative perceptions of healthcare spaces. Additionally, LGBTQIA2+ patients also experience access issues related to the social determinants of health, such as housing and food insecurity, unemployment, insurance coverage issues, discrimination related to sexual orientation and gender identity, and mental health issues [3]. These issues further increase health disparities among this population.

Negative perceptions of healthcare can contribute to poor health outcomes in LGBTQIA2+ patients. Patients who reported higher levels of health-related stereotype threats were associated with delays in scheduling mental health appointments and poorer self-reported mental health outcomes [4]. Furthermore, a qualitative study on LGBTQIA2+ patient experiences in clinical spaces highlights how severe these negative experiences for LGBTQIA2+ patients are, including providers violating confidentiality and using the wrong pronouns [5]. While many of these instances were viewed as unintentional, these inadvertent microaggressions still impact patient experiences negatively. As a result, LGBTQIA2+ patients may avoid annual checkups and routine cancer screenings [6], highlighting the detrimental impact that negative perceptions due to microaggressions have.

In addition to the issues of creating safe clinical spaces for LGBTQIA2+ identifying patients, other factors like socioeconomic status and other social determinants of health can negatively affect LGBTQIA2+ patients’ access to comprehensive healthcare. In the USA, LGBTQIA2+ identifying patients are more likely to be uninsured and less likely to have a personal healthcare provider compared to non-LGBTQIA2+ patients, with trans patients and queer patients being more likely to be uninsured [7], suggesting severe socioeconomic barriers to accessing care [8]. There can be many explanations for why LGBTQIA2+ patients might be more likely to be uninsured including losing access to health insurance due to familial rejection [7]. A lack of insurance and the overall cost of healthcare can be financially inhibitory, causing LGBTQIA2+ patients to delay or even avoid care, leading to worse health outcomes, especially when it comes to regular cancer screenings [9].

LGBTQIA2+ patients are rarely included in clinical trials through a lack of inclusion in study protocols, informed consent, and trials not addressing their needs and demographics [10]. Not including the LGBTQIA2+ community means we are not learning about interventions or treatments' efficacy and safety in our LGBTQIA2+ patients [10]. There is a paucity of data and literature on LGBTQIA2+ patients in clinical trials, even after the NIH (National Institutes of Health) and FDA (Food and Drug Administration) granted funding for vulnerable populations; out of 71 manuscripts published, only 2 mentioned LGBTQIA2+ [11]. Specifically in cancer trials, very few have included LGBTQIA2+ patients meaning we know less about the impact of new treatments, side effects, and specific needs for our LGBTQIA2+ patients [12].

## Case study – Rainbow Clinic

### Rainbow Clinic background and description

Many clinical institutions have created LGBTQIA2+-specific clinics; however, few have successfully developed a free clinic dedicated to LGBTQIA2+ patients [13]. A mid-western University-associated student-run free clinic created a free LGBTQIA2+ clinic (the Rainbow Clinic) aimed to provide primary care healthcare services for LGBTQIA2+ identifying patients. The Rainbow Clinic was founded by a biomedical engineering undergraduate student and faculty champion from the Midwestern University’s College of Nursing. The Rainbow Clinic is entirely student-run and was created because of several issues underinsured and uninsured LGBTQIA2+ patients experience to foster a safe and inclusive space for patients to receive healthcare services. This clinic is important in addressing healthcare accessibility issues for LGBTQIA2+ patients that are often caused by disparities in their social determinants of health.

Prior to the formation of the Rainbow Clinic in April 2022, microaggressions were observed by several clinical volunteers in the general student-run free clinic, including deadnaming and incorrect pronoun usage, causing discomfort for LGBTQIA2+ patients. The Rainbow Clinic occurs bimonthly and was held in June, August, October, and December of 2022, as well as February, April, and May of 2023. The Rainbow Clinic is in a mid-western urban area that predominantly serves uninsured and underinsured, working class, and ethnically diverse patients and operates on Thursdays in a university-associated family medicine clinic from 5 to 10pm. The Rainbow Clinic is exclusively staffed by volunteer nurse practitioner and medical students with an interest in LGBTQ + health. Medical students who are members of the LGBTQ + interest group are emailed about volunteer opportunities. Nurse practitioner students are informed about volunteer clinical opportunities during their clinical didactic course. All students are precepted by medical providers from surrounding healthcare institutions, providing comprehensive primary care, gynecological and urological care, sexually transmitted infections (STI)/human immunodeficiency virus (HIV) testing, mental health resources, medication refills, diagnostic imaging, and laboratory services.

### Rainbow Clinic patient demographics

Through May of 2023, 85 patients signed up for appointments over the 7 clinics that were hosted; however, 36 patients either canceled or did not appear for their appointments, resulting in 49 patients being seen. Patients were recruited through social media platforms like Instagram™ as well as through word of mouth from community partners and institutions. The reported data are based on optional pre-appointment and post-appointment survey completion, not on patient registration data, resulting in less patients completing the survey compared to patients seen. Based on optional intake surveys that 33 patients completed, patient ages ranged from 18 to 36, with an average age of 25 (24.6). Patients were asked to self-identify their sexual orientation, gender identity, and racial ethnicity and were allowed to select more than one option. The largest percentage of patients surveyed identified their sexual orientation as bisexual (41.18%), followed by Gay (26.47%), Pansexual (11.76%), Lesbian (8.82%), Demisexual (5.88%), and Other (5.88%). Self-identified gender identity revealed that the largest percentage of patients identified as cis-female (30.56%), followed by cis-male (22.22%), non-binary (13.89%), trans-male (11.11%), other (11.11%), two-spirit (5.56%), and genderqueer (5.56%). Self-identified racial ethnicity showed that the largest percentage of patients identified as White/Caucasian (61.54%), followed by Black or African American (20.51%), Other (7.69%), Asian (5.13%), Hawaiian/Pacific Islander (2.56%), and Native American (2.56%).

### Rainbow Clinic satisfaction and comfortability results

Prior to and after appointments, patients at the Rainbow Clinic are asked to complete a survey that aims to evaluate both patient satisfaction as well as perceived comfortability in clinical spaces. Up to the most recent clinic in May of 2023, 33 patients filled out the pre-appointment survey and 31 patients filled out the post-appointment survey. As this was a voluntary survey, not all patients that completed the pre-appointment survey also completed the post-appointment survey. This tool was investigator-created and validated through peer review from clinic providers and researchers. Patients were asked questions that required either a Likert score (1–5), ranging from strongly disagree to strongly agree, as well as yes or no responses. The mean values of Likert-scored questions were evaluated with a two-tailed *t* test and compared to a mean value of 3 and a standard deviation of 1, which would indicate an indifference towards disagree or agree.

**Figure 1.**
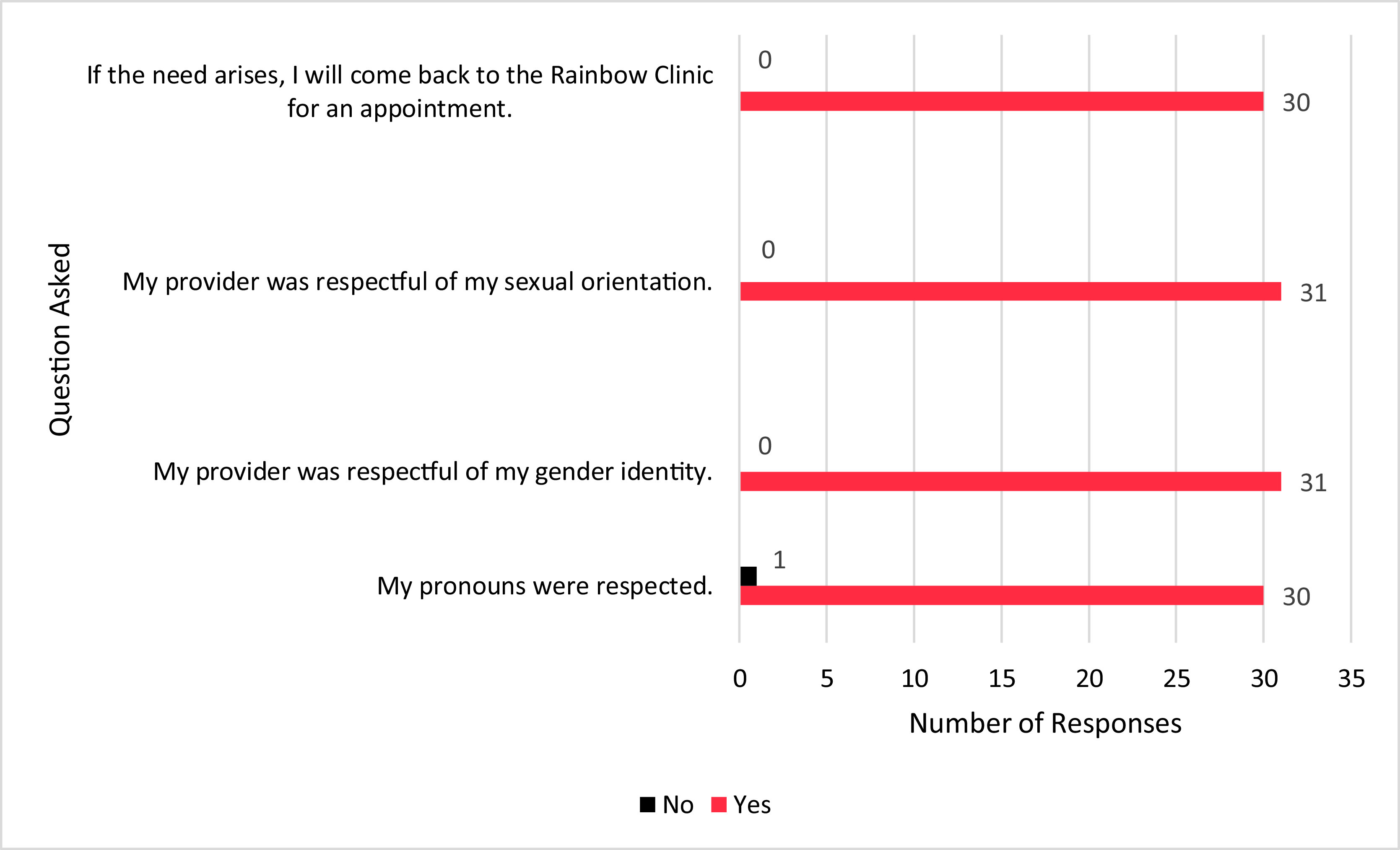
Post-Appointment Yes/No Questions and Responses

**Figure 2.**
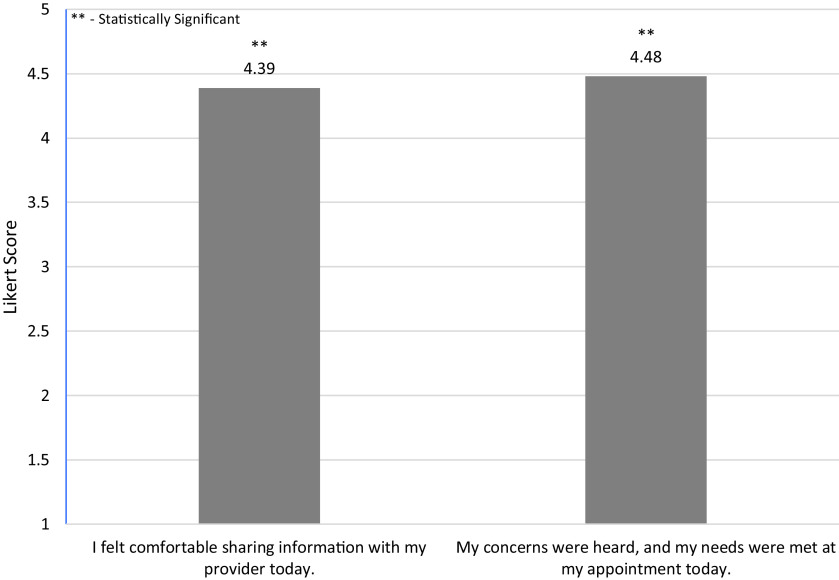
Post-Appointment Likert Survey Questions and Mean Responses

**Figure 3.**
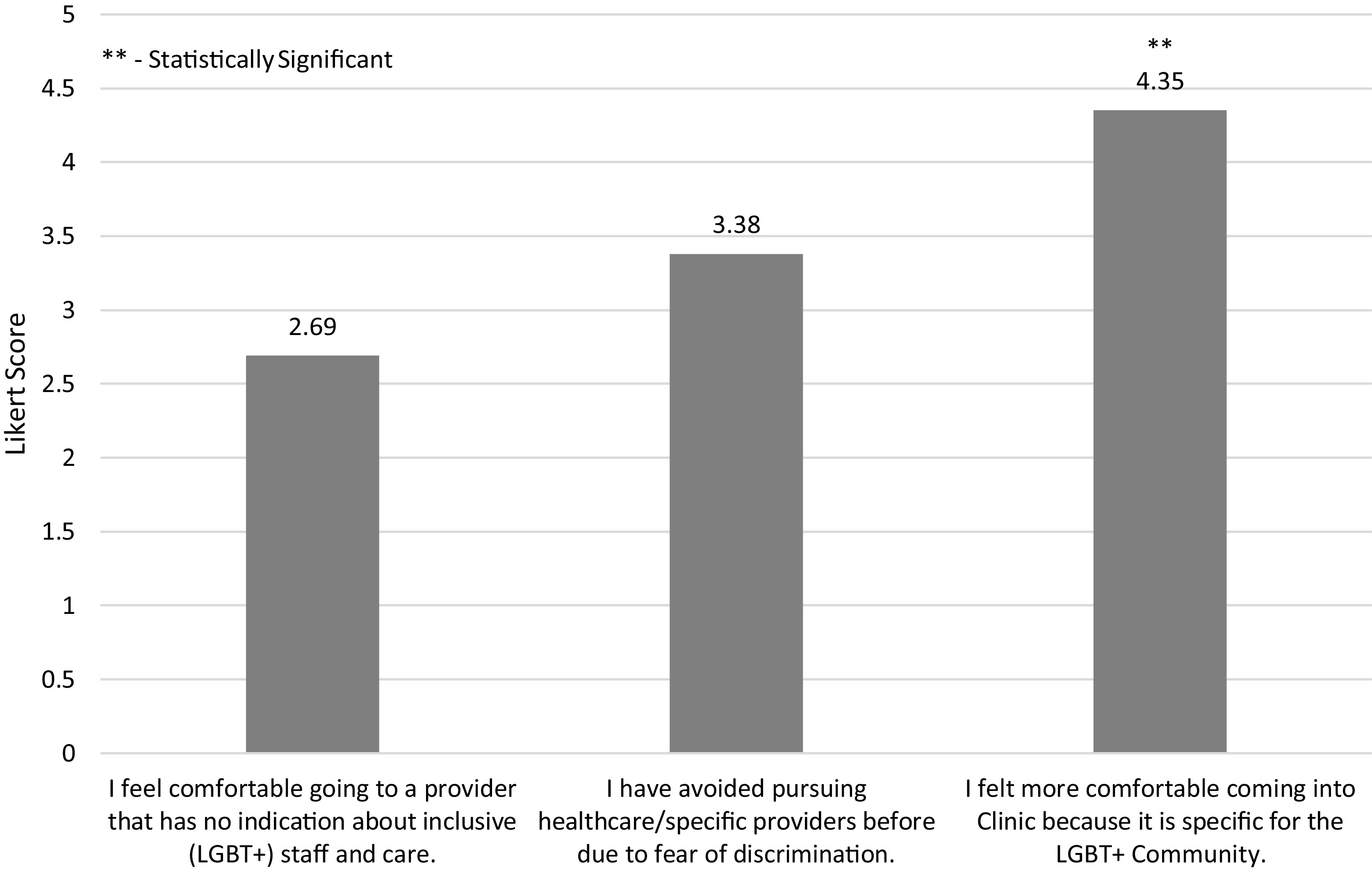
Pre-Appointment Likert Survey Questions and Mean Responses

Regarding patient experiences at the Rainbow Clinic, most patients felt their pronouns were respected, their provider respected their sexual orientation, and if needed they would visit the Rainbow Clinic again (Fig. 1). There was statistical significance (*p* < 0.0001) among patients in terms of their comfortability with sharing information with the provider and feeling their needs were heard and addressed (Fig. 2). Additionally, there was also statistical significance (*p* < 0.0001) among patients’ comfortability going to the Rainbow Clinic because it was specifically for the LGBT + community (Fig. 3).

## Case study evaluation

The Rainbow Clinic has established a safe space for the LGBTQIA2+ community. Based on the data and patient stories, this clinic has been a refuge for patients who have not gotten a cervical pap smear in 10 years or even those who have not seen or established a primary care provider at any point in their life. There needs to be more clinics specific to serving the LGBTQIA2+ community. This clinic respects the patient, listens to their concerns, and provides care to socioeconomically disadvantaged patients. The survey results also indicate that the intentional labeling of clinics as safe for LGBTQAI2+ patients is important in increasing the comfort for patients to receive care at these institutions. These clinics can also provide a space to conduct clinical trials. We know LGBTQIA2+ patients are underrepresented in clinical trials and having these well-established clinics will be a great place to educate and recruit patients.

## Dissemination and replication

This Rainbow Clinic was established utilizing the existing infrastructure of an already established student-run free clinic. Resources including facilities, protocols, and lab/pharmacy infrastructure were already in place prior to the establishment of the clinic, making the formation of the clinic simpler; however, there were several important considerations that were included to ensure the success of the Rainbow Clinic.

The Rainbow Clinic required all student and provider volunteers to complete LGBTQIA2+-specific training through the Fenway Institute. The faculty champion incorporated the Fenway Institute modules into their nurse practitioner didactic coursework, which were then applied to all students and providers volunteering at the Rainbow Clinic. In general, training can be developed by staff at specific clinical institutions to meet the more specific needs of the patient population being served; however, the Rainbow Clinic mandated that all undergraduate, graduate, and professional volunteers take several online modules from the Fenway Institute, which provides free online training modules that can also qualify for Continuing Medical Education credit. Training completion was checked by the undergraduate founder and stored for clinic records. These training courses, although perhaps more basic in nature, were important in educating and reminding staff of the best practices when it comes to interacting with LGBTQIA2+ patients, including the proper use of chosen names and pronouns. Clinical institutions interested in establishing an LGBTQIA2+-specific free clinic or clinical space should incorporate some aspect of trauma-informed education prior to setting up these spaces to avoid issues of deadnaming and other microaggressions that can negatively impact an LGBTQIA2+ patient’s experience.

Clinical testing and screening options were expanded to include more options for STIs and HIV testing. Prior to the establishment of the clinic, non-symptomatic STI testing was often referred to other local institutions that already offered free STI testing. The Rainbow Clinic recognized that many LGBTQIA2+ patients may come in for a chief complaint of STI testing but might have several health issues that could be related in addition to their need for STI testing. As a result, the Rainbow Clinic allows patients to be evaluated for non-symptomatic STI testing, which has allowed patients to address several issues related to their health at visits. Additionally, options for types of HIV testing were expanded as well. Initially, the clinic used a Western Blot test which could detect HIV infection 45–60 days after infection, but with the availability of the HIV antigen/antibody immunoassay that can detect HIV infection 15–20 days after infection, the clinic transitioned to immunoassay testing [14].

A consistent list of faculty providers for the Rainbow Clinic was developed. While there were only two instances of negative provider interactions at the Rainbow Clinic, one involving two providers and another involving a provider and a patient, it was important to establish a list of consistent, vetted, and Fenway-trained providers that were allowed to volunteer at the Rainbow Clinic. These providers would be individuals who have either already volunteered at the clinic or have been referred to the clinic by existing providers. Providers might also be chosen from existing LGBTQIA2+ clinical practices or departments that may work more intimately with LGBTQIA2+ patients such as infectious disease, OB-GYN, and family medicine. A list of vetted providers is important because the Rainbow Clinic had negative provider-patient interaction, where a provider came in a singular time, looking to gain volunteer hours from the clinic, but unfortunately mishandled a delicate patient case. Situations like this emphasize the importance of pre-training for any volunteers and must be avoided if possible because as mentioned previously, negative clinical experiences can further deter LGBTQIA2+ patients from seeking care.

## Future work

The next step of the Rainbow Clinic is to establish a free Pre-Exposure Prophylaxis (PrEP) clinic. The county where the Rainbow Clinic is located was deemed a high-risk area for HIV cases by the Health Resources and Services Administration. Therefore, providing free PrEP resources to the existing LGBTQIA2+ and non-LGBTQIA2+ patients is critical in reducing the spread of HIV and adhering to the goals of the national Ending the HIV Epidemic initiative. The Rainbow Clinic will be working with existing national programs to provide free oral PrEP medications and will provide all testing and clinical care at PrEP-specific Rainbow Clinics. Additionally, the Rainbow Clinic has been collaborating with several researchers at regional academic institutions to aid in patient recruitment for research being conducted at these institutions. The clinic aims to educate patients on clinical trials, engage in conducting clinical research, and provide data to patient registries.

## Conclusion

Among the more basic tasks such as securing a clinical space and providers, any effort to create safe, trauma-informed clinical space for LGBTQIA2+ patients is vital. While not all clinical institutions might be able to create free LGBTQIA2+ clinics, all clinical institutions can make active efforts to create safer spaces in their pre-existing clinics. Mandating basic training through institutions like the Fenway Institute can be a good step in making LGBTQIA2+ patients more comfortable to seek care, not only in LGBTQIA2+ health clinics but also in all clinical settings, leading to an increase in provider cultural competency and a decrease in negative health outcomes for the population.
